# Infections and Systemic Lupus Erythematosus: Binding or Sparring Partners?

**DOI:** 10.3390/ijms160817331

**Published:** 2015-07-29

**Authors:** Donato Rigante, Susanna Esposito

**Affiliations:** 1 Institute of Pediatrics, Università Cattolica Sacro Cuore, Fondazione Policlinico Universitario A. Gemelli, 00168 Rome, Italy; E-Mail: drigante@rm.unicatt.it; 2 Pediatric Highly Intensive Care Unit, Department of Pathophysiology and Transplantation, Università degli Studi di Milano, Fondazione IRCCS Ca’ Granda Ospedale Maggiore Policlinico, 20122 Milan, Italy

**Keywords:** systemic lupus erythematosus, Epstein-Barr virus, parvovirus B19, cytomegalovirus, retrovirus

## Abstract

Extensive work on experimental animal models clearly demonstrates that infectious agents can break immunological tolerance to self-antigens and induce autoimmune disorders, mainly systemic lupus erythematosus (SLE). The establishment of a causative link between infections and autoimmunity has been largely studied in a host of clinical studies, proving the role of infectious agents in the induction, as well as in the progression or exacerbation of SLE. However, we are far from a plain understanding of microbial-host interactions in the pathogenesis of SLE. Much serological, molecular and geoepidemiological evidence supports the relationship of different environmental infectious triggers in the inception of SLE-related autoimmune phenomena with adjuvant effects. The promotion of autoimmune responses through bystander activation or epitope spreading via multiple inflammatory pathways has been confirmed in animal models. Different viruses have been implicated in SLE pathogenesis, particularly Epstein-Barr virus, but also parvovirus B19, cytomegalovirus and retroviruses. SLE patients usually have an impaired immune response towards Epstein-Barr virus and dysregulation of the viral latency period. Furthermore, the accumulation of endogenous retroviral products might trigger the production of interferon and anti-DNA antibodies. In addition, protozoan infections might even protect from autoimmune processes and rescind an ongoing B cell activation. Herein, we discuss which type of infections induce, exacerbate or inhibit autoimmune disorders and analyze the principal infection-induced immunological mechanisms influencing the development of SLE.

## 1. Introduction

Autoimmunity is the result of self-tolerance breakdown and derives from an attack at the basis of the immune system on different organs and tissues, which are conceived of as foreign. However, the development of an overt autoimmune disorder requires the combination of immunologic, genetic and environmental factors, which might be of an infectious nature. Systemic lupus erythematosus (SLE) is the prototype of autoimmune disorders, caused by the effect of multiple polyclonal autoantibodies, which usually appear years before clinical manifestations are evident [1]. The onset might occur at whatever age, also in children, and approximately 20% of cases start in childhood with periods of flare and remission, resulting in irreversible tissue damage and even premature death [2].

At the molecular level, SLE is characterized by a persistent inflammatory state that is harmful for different organs, such as skin, joints, kidney, serous membranes, central nervous system and blood, with chronic inflammation resulting from both adaptive immunity dysregulation and hyper-production of different autoantibodies [3]. Diagnosis of SLE is based on the criteria established by the American College of Rheumatology in 1982 and the more recent systemic lupus international collaborating clinics’ classification criteria, drafted in 2012: these criteria include the presence of dermatologic signs, arthritis and serositis, variably combined with renal, neurologic and hematologic abnormalities, while additional diagnostic criteria are SLE-typical immunologic abnormalities, *i.e.*, anti-nuclear antibodies, doubled-strand DNA-directed autoantibodies, antibody to Sm nuclear antigen, anti-phospholipid antibody positivity, low complement and direct Coombs’ test [4,5].

In recent years, life expectancy for subjects with SLE has improved, and the actual 15-year survival rate is around 80% [6]. Pediatric SLE, starting at an age less than 16 years, is usually more aggressive than SLE in adults and often involves major organs, including kidneys and central nervous system. Furthermore, SLE in children is associated with increased mortality risk and reduced remission rates, while the major causes of death are severe infections following treatment with corticosteroids, immunosuppressive or biotechnological drugs [7].

The exact etiology of SLE is still unknown, but infections can act as environmental primers, inducing or promoting onset and exacerbations of SLE in genetically-predisposed individuals [8]. Induction of autoimmunity by viruses or bacteria is probably done by a “hit-and-run” mechanism when the causative agent has been cleared from circulation by the time of diagnosis. Nevertheless, despite intensive efforts, it remains difficult to identify a single microorganism as the unique cause of SLE, indicating that the “one organism-one disease” paradigm that is central to Koch’s postulates might not invariably apply to a microbiologically-related autoimmune disorder. Moreover, the exact interaction of these infectious triggers and their interplay with genes that confer susceptibility to autoimmunity is still poorly defined. For instance, it is unknown why some, but not all individuals go on to develop full-blown SLE symptoms with disease flares, and the reasons underlying relapses and remissions in SLE are unraveled.

## 2. A Baffling Alliance between Infections and Autoimmunity

An aberration of the physiological and protective processes of the immune system might occur during viral, bacterial, parasitic or fungal infections in genetically-predisposed subjects. Although genetic background confers susceptibility to disease onset, it is neither sufficient nor causative for SLE development. Several studies have suggested that the interaction of genetic and infectious factors may have a role in determining the outcome of an autoimmune process; however, this topic is still controversial, and further research is needed to better understand the dynamic interplay between genetics and infections. The role of environmental factors in the etiology of autoimmune disorders is suggested by the disease discordance rate between monozygotic twins.

During childhood, the developing immune system might be specifically vulnerable to external factors, such as infectious agents, while autoimmunity might be triggered through the cumulative effect of repeated infections [9]. The activation of autoreactive T and B cells is the potential result of different infectious diseases through the theory of “molecular mimicry”, a structural cross-reactivity between the pathogenic microorganism and human self-antigens [10]. A proof of causality between a mimic epitope and autoimmune disorders might rely on the active immunization of animal models utilizing autoreactive T cells or autoantibodies [11]. Acute rheumatic fever is the classical example of molecular mimicry-based post-infectious autoimmunity, and an association between previous streptococcal infections and rheumatic fever has been demonstrated for the majority of patients, with streptococcal epitopes, such as M-protein mimicking cardiac myosin and heart-derived peptides [12]. Alternatively, microbial products may induce autoimmunity by triggering the bystander activation of the immune system, with “epitope spreading”, *i.e.*, enhanced presentation of autoantigens by antigen-presenting cells and breakage of the anergic state, which leads to the expansion of dormant autoreactive clones.

In particular, in 2009, Zandman-Goddard *et al.* have shown a significant association between elevated antibodies against Epstein-Barr virus (EBV) and skin and joint symptoms in SLE patients and that exposure to EBV infection may predict a disease phenotype of mild SLE with cutaneous and articular manifestations and elevated titers of anti-Ro antibodies [13].

## 3. The Role of EBV in the Pathogenesis of SLE

EBV is a ubiquitous pathogen, notoriously associated with many autoimmune disorders, such as multiple sclerosis and rheumatoid arthritis, and certainly the environmental agent most closely associated with SLE: EBV infection is more common in patients with SLE than in the healthy population, suggesting that patients might have impaired EBV-specific immune responses [14]. Increased prevalence of anti-EBV-humoral response directed at nuclear (EBNA), viral capsid (VCA) and early antigens (EA) has been observed in SLE patients [15]. The interaction between EBV and SLE is bidirectional, as on the one hand, EBV may trigger autoimmune processes, but on the other hand, SLE patients show both dysregulated anti-EBV response and an abnormal viral latency period [16]. In particular, the EBNA-1 antigen contains regions with considerable homology to sequences of SLE-associated autoantigens, such as PPPGRRP or PPPGMRPP sequences with the ribonuclear protein Smith (Sm) antigen or the Ro self-protein [17].

During primary infection, EBV-infected autoreactive B cells proliferate and express virus-encoded anti-apoptotic molecules, becoming largely predominant in genetically-predisposed individuals and acting as antigen-presenting cells; T cells migrate to the target organs and receive a co-stimulatory survival signal from the infected B cells, where they proliferate and maintain a chronic inflammatory picture [18]. Different EBV antigens can exhibit either structural, molecular or functional mimicry with SLE antigens or other critical immune-regulatory components.

SLE-specific autoantibodies might arise from the immune reaction against EBV nuclear antigens, which cross-react with specific host’s autoantigens (Ro or Sm), molecular mimickers of EBV antigens, and indeed, anti-Ro autoantibodies are the earliest antibodies detected in the SLE preclinical period [19]. Some studies have reported a prevalence of 99% of EBV infection in young SLE patients, compared to 70% prevalence in control groups [20]. In addition, decreased CD8+ T cell and increased CD4+ T cell response to EBV have been demonstrated, suggesting a defective control of latent EBV infection, and the default of the T cell response to EBV was believed crucial to enable persistent latent viral disease [21].

EBV may finally cause a defect in B cell tolerance checkpoints, as shown in transgenic lupus mouse models by the induction of B cell activating factor of the tumor necrosis factor family (BAFF), also known as a B lymphocyte stimulator, a vital homeostatic cytokine for B cells that helps regulate both innate and adaptive immune responses [22]. SLE patients have abnormal expression of viral mRNAs in their peripheral blood mononuclear cells, indicating a redundant replication of the virus in comparison to healthy subjects, and such an altered infection pattern may contribute to the pathogenesis of SLE [23]. Immunization with EBNA-1 or EBNA-1-derived peptides has led to the development of SLE-like autoimmunity in mice, while rabbits immunized with the Ro 60 kD antigen or with EBNA-1 peptide having developed cross-reacting antibodies and even SLE typical manifestations, such as leukopenia, thrombocytopenia and nephritis [24,25,26].

## 4. The Relationship of Other Infectious Agents with SLE

In recent years, the occurrence of SLE has been associated with numerous viral infections, and antigenic cross-reactivity combined with molecular mimicry have been suggested as their basic foundation. An example for cross-reactivity is the peptide deriving from Coxsackie virus 2B protein, having 87% amino acids homology with the 222–229 region of the major linear antibody binding site of Ro 60 kD autoantigen [27].

Human parvovirus B19, the cause of fifth disease in childhood, has emerged as one of the main contributors to autoimmunity within the last few years, as it may simulate both clinical and laboratory features of SLE, presenting as a potential first-time diagnosis, as well as an exacerbation of a previously-established SLE: the similarities of clinical and serological features of parvovirus infection and SLE at onset may hinder the differential diagnosis between these two conditions. Effectively, parvovirus B19 infection mimicking SLE usually fulfils less than four American College of Rheumatology criteria for SLE, including, in rare cases, even cardiac or renal involvement and hemolytic anemia, variably associated with short-lived low titers of autoantibodies [28]. Pavlovic *et al.* have proposed a model for parvovirus B19 association with SLE, resulting from the secretion of hydrolyzing anti-ssDNA autoantibodies, which can hydrolyze viral B19 ssDNA in blood and other fluids, and from viral ssDNA translocation into the nucleus of the host permissive cells, contributing to the perpetuation and maintenance of a “vicious cycle” in SLE flares [29]. Some studies have postulated a connection between human parvovirus B19 infection and anti-phospholipid antibodies (APhL) [30]. B19 infection and anti-phospholipid syndrome (APS) exhibit congruent symptoms. Recently, phospholipase A2 (PLA2)-like activity has been linked to the VP1 unique region (VP1u) of B19. It has been observed that autoantibodies against cardiolipin (CL), beta-2-glycoprotein I (β2GPI) and phospholipid (PhL) in sera from patients with B19 infection were cross-reactive with B19-VP1u [31]. Consistently, sera from rabbits immunized with recombinant B19-VP1u protein displayed raised detectable immunoglobulins against B19-VP1u, CL, β2GPI and PhL [31]. Additionally, the mice immunized with anti-B19-VP1u IgG developed thrombocytopenia, prolongation of aPTT and autoantibody against β2GPI and PhL [31]. Furthermore, aggravated disease activity is also detected in lupus-prone mice that were treated with B19 viral protein [32].

Several studies have also demonstrated a correlation between cytomegalovirus (CMV) infection and SLE: the presence of both anti-CMV IgM and CMV-DNA has been detected at disease onset in some cases of SLE [33,34]. In particular, Stratta *et al.* found that CMV infection was significantly associated with the “vascular” manifestations of SLE, showing the risk of arterial damage, and less frequent typical histological renal pictures responsible for nephrotic syndrome [35].

Human endogenous retroviruses (HERVs) are fossil viruses that started to be integrated into the human genome about 30–40 million years ago and now make up 8% of the genome: they are believed to be pathogenic in several autoimmune disorders through molecular mimicry and immune dysregulation, and ERV-encoded proteins might effectively become targets of autoreactivity. Different antibodies to *gag* and *env* regions of HERVs have been reported in patients with autoimmune disorders, including SLE [36]. In addition, the 70 k/U1 small nuclear RNA (snRNP) is a human autoantigen that has homology and cross-reactivity with a p30 *gag* retroviral protein [37]. The human endogenous retroviral sequence HRES-1 is centrally located at the 1q42 chromosomal region, one of at least 10 chromosomal loci shown to be linked to the potential development of SLE and one of the most common fragile sites in the human genome: Pullmann *et al*. defined six haplotypes of the HRES-1 locus that influence the development of SLE and identified the *Hind*III/*Nci*I *C*/*C* allele as a predictor of renal disease and minimal lung involvement [38]. HTLV-1 and HIV-1 retroviruses have also been implicated in the pathogenesis of SLE: both dysregulated apoptosis and the shift from a T helper type 1 to a T helper type 2 cytokine profile have been demonstrated in SLE and HIV-infected patients [39].

The relationship between hepatitis C virus (HCV) and SLE has not yet been clearly established, though the prevalence of HCV infection was higher in SLE patients than in blood donors from the same geographic area in a 2000 study by Ramos-Casals *et al*.: moreover, SLE HCV-positive patients showed a lower frequency of cutaneous SLE features and anti-dsDNA antibodies, but a higher prevalence of liver involvement, hypocomplementemia and cryoglobulinemia [40]. Interestingly, antibodies to proliferating cell nuclear antigen (PCNA) have been demonstrated also in patients with chronic HCV infection, indicating that anti-PCNA antibody may not be specific for SLE [41].

An interaction between bacteria and SLE has been documented in animal models and humans, and elevated titers of anti-dsDNA antibodies can be detected in healthy individuals following different bacterial infections [42]. Moreover, anti-dsDNA antibodies derived from naive lupus-prone mice can react with both the cell surfaces of murine endogenous microbial flora and glycolipid components of the mycobacterial cell wall [43]. The presence of a bacterial infection triggers the immune system through specific products, such as bacterial lipopolysaccharides or nucleic acid-containing immune complexes [44]. Pathogen-associated molecular patterns (PAMPs) interact with toll-like receptors (TLRs) and non-TLR internal receptors of antigen-presenting cells, monocytes, B and T lymphocytes. The binding of TLRs induces plasmacytoid dendritic cells to release interferon, leading to the production of proinflammatory cytokines and destabilizing innate immunity processes [45]. Recent clinical studies have placed new emphasis on the role of TLRs, specifically TLR-7 and TLR-9, in the promotion of autoantibody production. Pharmacologic modulation of TLR-directed pathways might offer new additional therapeutic approaches for the treatment of SLE [46].

A concomitant APS and even macrophage activation syndrome (MAS) can be observed in SLE patients: APS is usually revealed by thrombotic manifestations associated with circulating anti-phospholipid antibodies, while MAS, also named secondary hemophagocytic lymphohistiocytosis, is heralded by multiorgan dysfunction, coagulopathy, pancytopenia and shock [47,48]. Many infections are accompanied by the presence of anti-phospholipid antibodies, and infections may simultaneously be present in patients with clinical manifestations of APS and SLE [49]. In addition, the outburst of MAS can be triggered by different microbes, and polymerase chain reaction search for viral nucleic acid sequences, including EBV, Parvovirus B19 or CMV, is recommended in SLE clinical settings characterized by non-remitting fever, organomegaly and hyperferritinemia [50].

Further prospective studies are justified to validate these correlations dealing with acute infections and specific SLE manifestations, with the aim of a better understanding of their mutual relationship.

## 5. The Protective Effect of Infectious Agents from Autoimmune Processes and SLE

Epidemiologic evidence suggests that the recently increased reports of autoimmune phenomena could be partly linked to the improvement of the socio-economic level for many Western countries, more specifically to the decrease of infections observed over the last few decades: this “hygiene hypothesis” postulated that the infection-related inflammatory reaction might protect rather than induce or accelerate an autoimmune process [51]. A host of studies have suggested that autoimmune disorders can be prevented or improved following microbial infections. For instance, several epidemiological studies have linked the disappearance of malaria with the increased reports of multiple sclerosis in Sardinia (Italy) [52].

The exact mechanism related to the protective effect of infections on autoimmune disorders is most likely multifactorial. Indeed, different pathogens have developed efficient methods to overcome adaptive immune mechanisms, and there is increasing evidence that they might insinuate their own anti-immune strategies into a susceptible vertebral host, by blocking antigen presentation, interfering with Toll-like receptors’ signaling, altering the cytokine milieu, which is crucial for an effective immune response, or by a mechanism of antigenic competition, which would induce a decreased response against self-antigens [53].

Parasitic infections may induce variable immunomodulatory effects [54]. *Toxoplasma gondii* is a ubiquitous single-celled intracellular protozoan that has recently been associated with autoimmune processes: Chen *et al.* demonstrated that *T. gondii* infection may prevent the progression of SLE-related nephritis in New Zealand black × New Zealand white (NZBW) F1 mice, as a consequence of downregulated intracellular expression of interferon-γ and interleukin-10 [55]. Fischer *et al.* evaluated the seroprevalence and clinical correlation of anti-*T. gondii* antibodies in European patients with rheumatoid arthritis and SLE, finding a higher prevalence of anti-*T. gondii* antibodies in those with rheumatoid arthritis than in SLE patients (63% *vs.* 36%, respectively), and that the rates of seropositivity of IgG against other infectious agents were comparable between the two groups [56].

Clatworthy *et al.* showed that FcγRIIb-deficient mice had increased clearance of plasmodia-causing malaria, finding that the inhibitory IgG receptor FcγRIIb is important in controlling the immune response to malarial parasites and suggesting that the higher frequency of human FcγRIIb gene polymorphisms might predispose to SLE in Asians and Africans, as these variants reduce patients’ susceptibility to malaria [57].

Furthermore, animal models provide evidence that various autoimmune disorders are suppressed in the course of long-term infections by parasitic worms, both Nematoda and Platyhelminthes, following a manipulation of the immune response towards a Th2-like phenotype, with modulation of Toll-like receptors’ signaling and secretion of anti-inflammatory cytokines, such as interleukin-10 [58].

*Helicobacter pylori*, a spiral Gram-negative bacillus that colonizes the human stomach and plays a key role in the pathogenesis of a number of gastroduodenal diseases, has been also related to different non-gastrointestinal disorders. Sawalha *et al.* investigated the prevalence of *H. pylori* seropositivity as part of a larger serologic survey in a cohort of 466 patients with SLE, finding a low rate of specific *H. pylori* antibodies and suggesting that this pathogen might exert a protective role on the risk of developing autoimmune disorders [59].

Ram *et al.* aimed to determine the presence of hepatitis B core antibody, a seromarker for past or present infection with hepatitis B virus (HBV), in a large number of sera collected from patients with different autoimmune disorders, but found an unexpected low percentage of specific antibodies in patients with SLE in comparison to healthy blood donors: this finding elicited the hypothesis of HBV’s protective role for autoimmunity [60].

## 6. What about Immunizations and SLE?

Last, but not least, some recent reports have associated the triggering effect of vaccinations with SLE: the existing data do not link vaccines and autoimmune phenomena directly in a causal relationship; nevertheless, a temporal connection has been hypothesized in some reports, and transient increases in different autoantibodies have been reported after immunizations.

The question of a link between vaccinations and autoimmune disorders is surrounded by controversies, and vaccines against measles, tetanus, influenza and HBV have been variably related to events ranging from production of autoantibodies to full-blown autoimmune disorders. The autoimmune/inflammatory syndrome induced by adjuvants (ASIA) is one example where vaccinations and heavy metals have been implicated as triggers of autoimmunity, even in children [61]. The mechanism of an autoimmune process following immunization has not yet been elucidated, but one of the possibilities is again molecular mimicry with self-antigens [62]. An international case-control study conducted between April 2008 and June 2012 involving SLE patients, diagnosed according the American College of Rheumatology criteria, assessed their vaccination status and found no association between exposure to vaccinations and risk of developing SLE [63].

The quadrivalent human papilloma virus vaccine has been considered safe and well tolerated in a series of adolescents and young women with SLE, with no increase in mean Systemic Lupus Erythematosus Disease Activity Index (SLEDAI) scores [64].

Agmon-Levin *et al.* raised concerns about HBV vaccination for patients with SLE. They examined the effects of immunization with HBV vaccine in a murine model at eight and 12 weeks of age: the vaccination induced high anti-dsDNA antibodies, acceleration of kidney disease revealed by increased proteinuria and deposition of HBs antigen in the kidney, so that the authors concluded that different components of the vaccine might be linked to adverse effects of an autoimmune nature [65]. However, no similar data have been collected in humans.

## 7. Conclusions

The mosaic of autoimmunity has still many lacking tesserae: in particular, the etiopathogenesis of SLE remains far from being completely elucidated. Environmental and genetic factors have been implicated in the induction and progression of this disease. Much attention has been given to genetic research on the path to uncovering the underlying factors of autoimmunity, and many genome-wide association studies have identified numerous gene-disease associations for SLE, though the maintenance of autoaggressive responses and the development of clinically-overt disease have not been yet delineated. The concept of molecular mimicry as a central mechanism by which an autoimmune process is induced is largely known for SLE, but we have not yet unveiled the molecular aspects linking the environment to genetic susceptibility factors and immune dysregulation. Among infections, particularly EBV, Parvovirus B19, retrovirus and CMV infections might play a pivotal role in the starting point of autoimmune processes. The multi-faceted interactions between infections and autoimmunity reveal many possibilities for either causative or protective associations. Indeed, some infections (primarily protozoan infections) might confer protection from autoimmunity, depending on the unique interaction between the microorganism and host. There is a need to corroborate these observations in animal models, to call for further studies to conclude that infectious agents are indeed one of the causes of SLE and to enable the design of novel specific therapies, as well as to examine the percentage of patients that exhibit autoimmune reactions to vaccines.
